# Rapid Construction of Liquid-like Surfaces via Single-Cycle Polymer Brush Grafting for Enhanced Antifouling in Microfluidic Systems

**DOI:** 10.3390/mi15101241

**Published:** 2024-10-09

**Authors:** Feng Wu, Jing Xu, Yuanyuan Liu, Hua Sun, Lishang Zhang, Yixuan Liu, Weiwei Wang, Fali Chong, Dan Zou, Shuli Wang

**Affiliations:** 1 School of Physics and New Energy, Xuzhou University of Technology, Xuzhou 221018, China; ztaiyang1@163.com (L.Z.); 20210802206@xzit.edu.cn (Y.L.); wwwang@xzit.edu.cn (W.W.); flchong2008@163.com (F.C.); 2 Medical Laboratory Department, The First People’s Hospital of Xuzhou, Xuzhou 221116, China; xujingwlc@outlook.com; 3 School of Material and Chemical Engineering, Xuzhou University of Technology, Xuzhou 221018, China; lyy8email@163.com (Y.L.); iamsunhua@xzit.edu.cn (H.S.); 4 School of Health Management, Xihua University, Chengdu 610039, China; zoudan@mail.xhu.edu.cn; 5 Fujian Engineering Research Center for Solid-State Lighting, Department of Electronic Science, School of Electronic Science and Engineering, Xiamen University, Xiamen 361005, China

**Keywords:** liquid-like surface, slippery, antifouling, microfluidics

## Abstract

Liquid-like surfaces have demonstrated immense potential in their ability to resist cell adhesion, a critical requirement for numerous applications across various domains. However, the conventional methodologies for preparing liquid-like surfaces often entail a complex multi-step polymer brush modification process, which is not only time-consuming but also presents significant challenges. In this work, we developed a single-cycle polymer brush modification strategy to build liquid-like surfaces by leveraging high-molecular-weight bis(3-aminopropyl)-terminated polydimethylsiloxane, which significantly simplifies the preparation process. The resultant liquid-like surface is endowed with exceptional slipperiness, effectively inhibiting bacterial colonization and diminishing the adherence of platelets. Moreover, it offers promising implications for reducing the dependency on anticoagulants in microfluidic systems constructed from PDMS, all while sustaining its antithrombotic attributes.

## 1. Introduction

Recent advancements in polydimethylsiloxane (PDMS)-based microfluidics have significantly transformed various fields, including biomedical engineering, chemical analysis, and materials science [1,2,3,4,5,6]. Central to the performance and functionality of these microfluidic systems is the interface, which plays a critical role in determining device efficiency [7,8]. A major challenge in microfluidics is fouling [9,10,11], which can severely impact system reliability and efficiency. This issue is particularly pronounced in in vitro blood testing, where the non-specific adhesion of non-target cells can compromise the subsequent cell analysis and identification [12]. Additionally, the use of soluble anticoagulant drugs, such as heparin, during detection processes can alter the biological characteristics of target cells, potentially affecting cell viability and protein expression [13,14]. Consequently, developing PDMS-based microfluidic chips with effective antifouling properties is of paramount importance.

To address the challenges of fouling in microfluidic systems, researchers have explored various antifouling strategies, including solid surface design [15,16] and liquid surface design [17,18]. Solid surface design involves techniques such as superhydrophobic or superoleophobic coatings, which create a physical barrier against cell adhesion [19,20]. This barrier reduces the likelihood of direct interactions between cells and the surface, thereby minimizing cellular attachment. However, the complex nanostructures required for these coatings can complicate their integration into microfluidic chips. In contrast, liquid surface design entails applying functional liquids to PDMS surfaces, creating extremely slippery interfaces that reduce cell adhesion [21,22]. This approach offers several advantages, including resistance to bacterial colonization, decreased macrophage penetration, and a reduced inflammatory response. Despite these benefits, liquid surface designs face challenges such as potential depletion and instability due to the inherent mobility of the liquid [23]. These limitations affect the practical application of both solid and liquid surface designs in achieving effective antifouling properties in PDMS-based microfluidic chips.

Recent research has underscored the exceptional properties of flexible polymer brushes, particularly bis (3-aminopropyl)-terminated PDMS (μPDMS), which exhibits an extraordinarily low glass transition temperature [1,24]. The chemical bonds within these brushes exhibit a notably low rotational conformation transition energy barrier, comparable to the energy present in thermal motion [25]. This characteristic endows μPDMS brushes with remarkable mobility at room temperature, resulting in a distinctly slippery “liquid-like” behavior. Such liquid-like surfaces have demonstrated considerable potential in various applications, including lossless or directional liquid transfer [26], resistance to fouling [25,27], and anti-icing [28] properties. In our recent work, we introduced a liquid-like surface functionalization strategy by grafting μPDMS in green solvent, which not only effectively resists cell adhesion but also exhibits superior biocompatibility, and realizes high-purity and high-efficiency circulating tumor cell (CTC) isolation in PDMS-based microfluidic chips [1]. However, the conventional preparation of this surface typically requires a multi-step modification process, which is both time-consuming and complex. Therefore, there is an urgent need to develop simplified methods to expedite the functionalization process, which is crucial for advancing the use of liquid-like surfaces in PDMS-based microfluidic systems.

In this paper, we established the rapid, liquid-like interfacial modification of PDMS surfaces by the single-cycle grafting of high-molecular-weight μPDMS brushes. The sliding performance of the modification process was better than the liquid-like surfaces with a four-cycle grafting of low-molecular-weight μPDMS brushes, demonstrating rapid and efficient functionalization. The flexibility of the modified μPDMS endow the liquid-like surfaces with an excellent antifouling performance for bacteria, platelets, and blood cells. Finally, we introduced the liquid-like interfacial design in the PDMS microfluidic channels and demonstrated its application prospects in anticoagulation.

## 2. Materials and Methods

The quartz crystals were obtained from Jiaxing Crystal Electronics Co. (Jiaxing, China). μPDMS with the molecular weights of 27,000 and 2500, N,N′-disuccinimidyl carbonate (DSC), and 3-aminopropyl triethoxysilane (APTES) were purchased from Sigma-Aldrich (St. Louis, MI, USA). Deionized water with a resistivity of 18.2 MΩ·cm was obtained from a Milli-Q system.

The ethanol solutions of DSC (1 μg mL^−1^) and μPDMS (molecular weight of 27,000, 1 μg mL^−1^) were prepared for the grafting of liquid-like polymer chains. The amino-functionalized substrates were immersed in the DSC ethanol solution for 60 min at room temperature, followed by rinsing with ethanol and rapid washing. Subsequently, the substrates were immersed in the μPDMS ethanol solution for an additional 60 min at room temperature, rinsed with ethanol, and then dried with compressed air. This process was repeated for several cycles when using the μPDMS with a molecular weight of 2500, and the concentration of the reactants and reaction conditions were without change.

For preparing the liquid-like surface-modified microfluidic inner surface, PDMS (Sylgard 184, Dow Corning Corporation (Midland, AL, USA), mixed at 10:1 base/cross-linker ratio) is first cast into single-channel molds and cured at 80 °C for 3 h. Subsequently, the PDMS was peeled off from the molds. The PDMS and the glass slide were treated with oxygen plasma to form silanol groups on the surface. Then, they were sequentially immersed in the ethanol solutions of APTES, DSC, and μPDMS and reacted at room temperature for 60 min. Finally, the PDMS and the glass slide were fixed together using dovetail clips to form the liquid-like surface-modified PDMS-based microfluidic chips.

The slippery property of the surface was measured by the tilted plate method. Briefly, a 10 μL blood droplet was placed on the surface. Subsequently, the surface was tilted relative to the horizontal plane until the blood droplet began to roll off the surface. The inclined angle can be considered the blood droplet sliding angle.

The density of amine groups on the samples was quantified using the acid orange II (AO II) colorimetric method [29]. In this procedure, the samples were first immersed in an aqueous solution of AO II at pH 4.0 for 4 h to enable reaction with the amine groups. Following this, the samples were thoroughly washed with a pH 4.0 aqueous hydrochloric acid solution to remove any unreacted AO II. Subsequently, the AO II bound to the samples was eluted using 200 μL of a sodium hydroxide solution at pH 11.0. The amount of eluted AO II was then measured by fluorescence spectroscopy, with excitation at 485 nm and emission at 520 nm, utilizing a Molecular Devices SpectraMax ID5 (San Jose, CA, USA). The amine group density was calculated by comparing the fluorescence data to a standard curve.

The amount of μPDMS grafted onto the surface was monitored using a Quartz Crystal Microbalance with Dissipation (QCM-D, Stockholm, Sweden). Initially, gold-coated quartz crystals were functionalized with DSC; subsequently, an ethanol solution of μPDMS was injected into the same cell. All the measurements were performed at room temperature to ensure consistent conditions throughout the experiment.

The chemical composition of the liquid-like polymer layer was analyzed using X-ray photoelectron spectroscopy (XPS). The measurements were performed with a PHI Quantum 2000 Scanning ESCA Microprobe (Physical Electronics, Eden Prairie, MN, USA), equipped with a monochromatic Al Kα X-ray source (1486.6 eV), operating at 15 kV and 35 W under a vacuum pressure of 5 × 10^−7^ Pa. The carbon peak at 284.4 eV was used as the reference for charge calibration.

The antibacterial activity of the liquid-like surface was assessed following the ISO 22196:2011 standard [30]. *Staphylococcus aureus* and *Escherichia coli* were precultured and diluted to 1/500 and 1/100, respectively, with nutrient broth to obtain a test inoculum with a concentration of approximately 6 × 10^5^ cells mL^−1^. On a sterile bench, sterilized PDMS plates were placed in a 24-well plate, and 100 μL of the diluted bacterial solution was added dropwise to the surface of each sample. Then, a clean PE film (Wuxi City, China) was used to cover the samples, which were then incubated in a 37 °C incubator for 24 h. Subsequently, a pipette was used to repeatedly pipette to completely detach the bacteria from the PDMS and the PE film. Finally, 100 μL of bacterial solution was drawn and evenly coated on the previously solidified solid medium and incubated at 37 °C for 24 h.

A dose of 10 U/mL heparin was added to fresh whole blood obtained from a volunteer. This heparinized blood was then applied to the sample surfaces and incubated for 1 h. Following incubation, the adherent blood cells were fixed with 2.5% glutaraldehyde solution at 4 °C for 2 h. The morphology of the adhered blood cells was subsequently examined using a scanning electron microscope (SEM, Hitachi S-2400, Tokyo, Japan).

Fresh whole blood, containing heparin at a concentration of 0.25 U/mL, was injected into the microfluidic chip at a flow rate of 0.1 mL/h. After allowing for 2 h, the PDMS and glass slide were removed, and the adherent blood cells on the chip were fixed with 2.5% glutaraldehyde solution at 4 °C for 2 h. The morphology of the adhered blood cells was then analyzed using a scanning electron microscope (SEM, Hitachi S-2400, Tokyo, Japan).

The data were expressed as mean ± standard deviation (SD), and all the experiments were repeated 3 times (mean ± SD, *n* = 3).

## 3. Results

**Figure 1:**
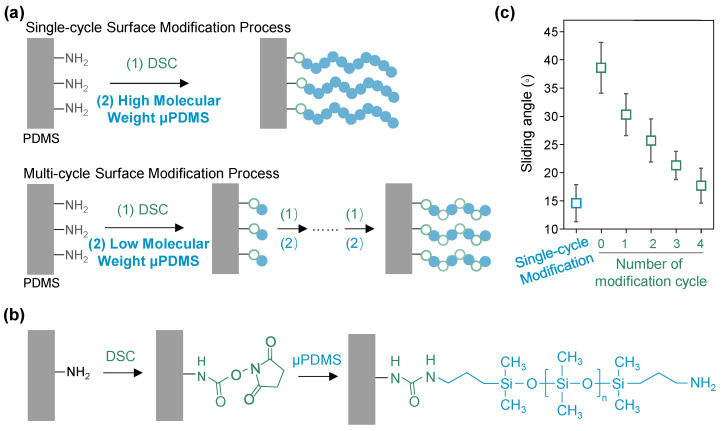
(**a**) The schematic of the preparation processes for liquid-like surfaces from the single-cycle grafting of high-molecular-weight μPDMS and multi-cycle grafting of low-molecular-weight μPDMS. The grass green hollow spheres denote DSC molecules, and the blue solid spheres represent μPDMS molecules. (**b**) The involved chemical reactions during the grafting of DSC and μPDMS on the APTES-modified PDMS surface. (**c**) The sliding angles of a 10 μL blood droplet on the liquid-like surfaces prepared from the single-cycle grafting of high-molecular-weight μPDMS, and 0–4 cycle grafting of low-molecular-weight μPDMS (mean ± SD, *n* = 3).

The schematic of the grafting process of the μPDMS for preparing liquid-like surfaces is illustrated in Figure 1a. The utilization of high-molecular-weight μPDMS facilitates a rapid functionalization process through a single-cycle modification approach. In contrast, employing low-molecular-weight μPDMS necessitates a multi-cycle modification process involving several reaction steps, thereby extending the overall preparation time for the liquid-like surfaces.

Figure 1b shows the chemical reaction that occurs in the single-cycle modification process for constructing the liquid-like surface. Initially, a monolayer of amino groups is grafted onto the PDMS surface via oxygen plasma treatment, followed by immersion in an ethanol solution containing APTES. Subsequently, the amino-modified surface is treated with an ethanol solution of DSC and μPDMS, facilitating the formation of liquid-like polymer chains. This process involves a ring-opening reaction between the succinimide groups and the amino groups on the PDMS surface [31]. This straightforward modification process enables the rapid functionalization of the liquid-like surface within a single processing cycle.

The μPDMS polymer brushes modified on the PDMS surface exhibit high mobility at room temperature due to their extremely low glass transition temperature, resulting in a highly melted state that imparts a liquid-like slippery property. To characterize this property, we measured the sliding angle of blood droplets (10 μL) on the liquid-like surfaces prepared from both the μPDMS with high and low molecular weight. As shown in Figure 1c, the blood droplet has a sliding angle of only 14.6° on liquid-like surfaces prepared through the single-cycle grafting of high-molecular-weight μPDMS. In contrast, the liquid-like surfaces modified from the single-cycle grafting of low-molecular-weight μPDMS exhibit a sliding angle of 30.3°. Increasing the grafting cycles of the low-molecular-weight μPDMS to four resulted in the reduction in the sliding angle to 17.7°. Thus, using the high-molecular-weight μPDMS allows for the rapid functionalization of the liquid-like surface, providing superior slipperiness and enhancing the antifouling performance.

**Figure 2:**
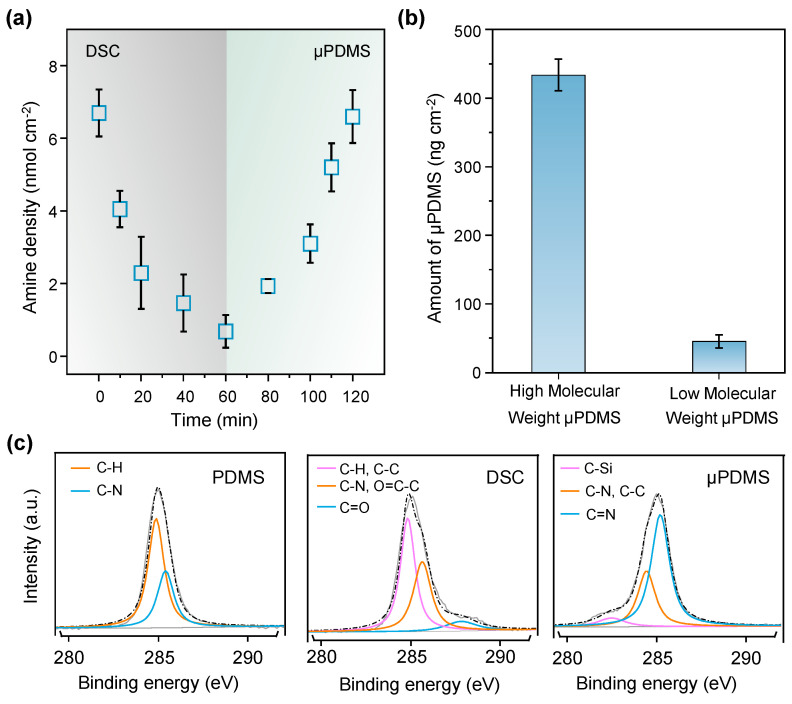
Physicochemical characterizations during the modification processes of liquid-like surfaces. (**a**) The amount of amine groups was determined by the acid orange method during the DSC and μPDMS grafting of 60 min. (**b**) The amount of μPDMS on the liquid-like surfaces prepared from the one-cycle grafting of high- and low-molecular-weight μPDMS (mean ± SD, *n* = 3). (**c**) The fitting curve of the peaks of C1s of XPS measurement before and after the modification of DSC and μPDMS.

The change in amino density on the PDMS surface offers a precise method for monitoring the reaction process during modification. As shown in Figure 2a, after the modification of DSC for 60 min, the surface amino density decreased from 6.72 nmol cm^−2^ to a minimum of 0.68 nmol cm^−2^. Following the subsequent μPDMS modification for the same duration, the amino density nearly recovered to its original value (6.61 nmol cm^−2^), indicating a relatively complete reaction and justifying the choice of 60 min as the optimal reaction time. To further quantify μPDMS grafting, we employed QCM-D for a single-cycle modification of 60 min. As shown in Figure 2b, the quantification results reveal that the high-molecular-weight μPDMS achieved a grafting amount of 433.6 ng cm^−2^, whereas the low-molecular-weight μPDMS was only 45.3 ng cm^−2^, approximately 10% of the former. This suggests successful μPDMS modification and demonstrates that the high-molecular-weight μPDMS yields longer polymer brushes within the same time frame. Long enough chains for the polymer brushes are one of the essential conditions for the construction of liquid-like surfaces. Therefore, the utilization of μPDMS with high molecular weight enables the rapid functionalization of the liquid-like surfaces. The surfaces’ chemical properties after each modification step were confirmed using XPS. For the PDMS modified with APTES, the high-resolution C1s spectrum could be fit into the peaks of C-H at 284.9 eV and C-N at 285.3 eV. After being modified with DSC, the characteristic peak of C=O at 287.8 eV appeared, which provided evidence of successful modification with DSC. Finally, when μPDMS modified PDMS, the characteristic peak of C-Si at 282.4 eV appeared, which served as evidence of successful modification by μPDMS chains (Figure 2c).

**Figure 3:**
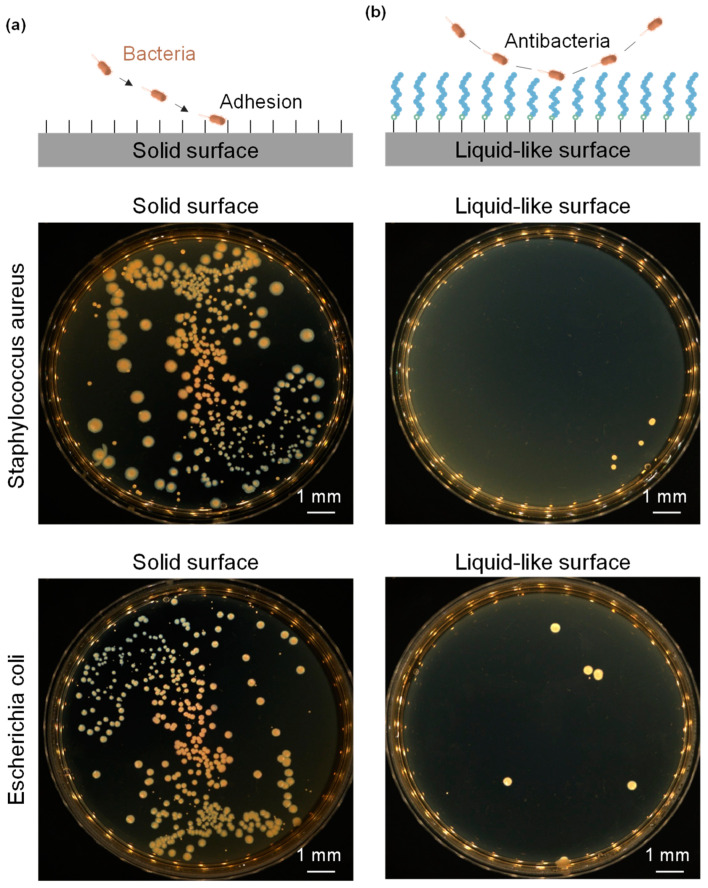
Colonization photographs of *Staphylococcus aureus* and *Escherichia coli* on (**a**) the solid surface and (**b**) the liquid-like surface.

Bacteria–material interactions are critical for applications requiring prolonged cellular proliferation in situ on the PDMS surface, as they may lead to bacterial adhesion and the formation of continuous bacterial biofilms [32], potentially compromising experimental accuracy [33]. The high flexibility of μPDMS imparts a liquid-like surface with reduced non-specific interaction forces for bacteria. This property renders the liquid-like surfaces highly promising for antibacterial applications. To assess these properties, we tested the interactions with *Staphylococcus aureus* and *Escherichia coli* as representative bacterial strains. As shown in Figure 3a, the solid surface is covered with a high density of bacterial colonies. In contrast, as depicted in Figure 3b, the colony density in the medium corresponding to the liquid-like surface is significantly lower than that corresponding to the solid surface. These findings indicate that the liquid-like surface possesses excellent antibacterial properties.

**Figure 4:**
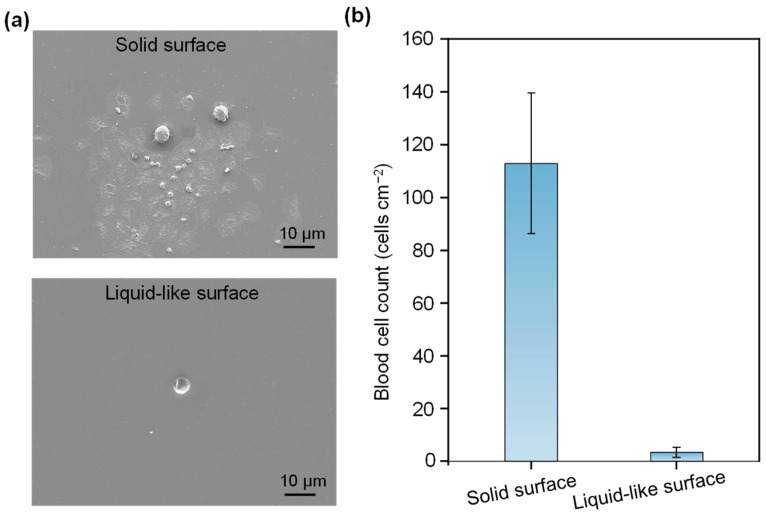
Adhesion of blood cells on solid and liquid-like surfaces. (**a**) SEM characterizations; (**b**) the number of adhered blood cells on both surfaces (mean ± SD, *n* = 3).

Blood cell–material interactions are pivotal in applications involving blood contact with PDMS-based materials, as they can lead to platelet adhesion and the subsequent thrombus formation [34,35,36]. Notably, the liquid-like surface has shown reduced interactions with platelets due to the flexible nature of μPDMS, suggesting a potential for minimizing platelet adhesion. To evaluate this, we conducted whole blood adhesion experiments to compare platelet adhesion on solid and liquid-like surfaces. As depicted in the SEM images in Figure 4a, a significant number of highly activated platelets adhere to the solid surfaces. In contrast, only a minimal quantity of platelets was observed on the liquid-like surfaces, indicating a substantial reduction in the non-specific platelet adhesion. The liquid-like surfaces, resembling a smooth and slippery surface, effectively repel platelets, preventing their easy adherence. Statistical analysis further demonstrates that platelet adhesion on the liquid-like surface was only 3% of that observed on the solid surfaces (Figure 4b).

**Figure 5:**
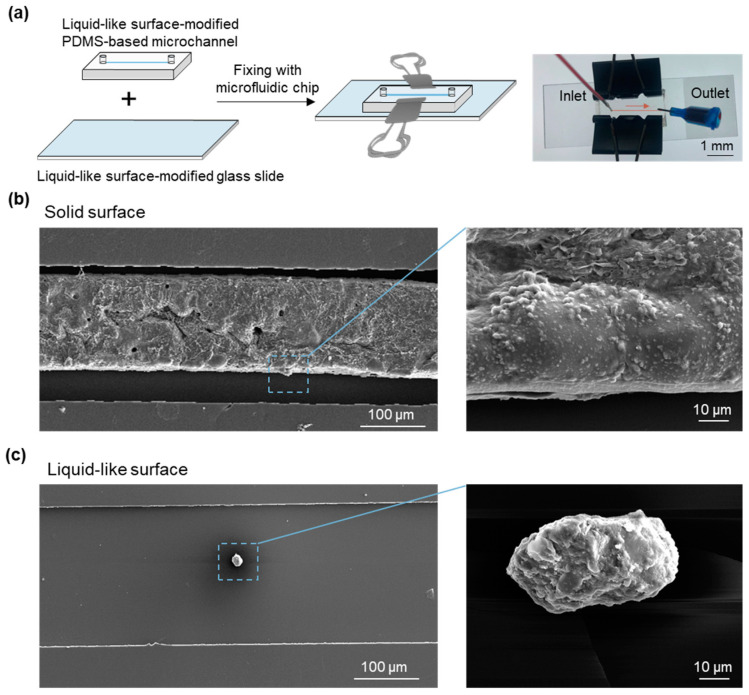
(**a**) Schematics of a closed microfluidic chip formed by fixing the PDMS and the glass slide together with dovetail clips and an image of the microfluidic chip after injection. Arrow shows the flow direction. The overall (left) and locally magnified views (right) of the SEM images of the microfluidic channels with (**b**) solid and (**c**) liquid-like interfacial design after the injection of fresh human blood.

The liquid-like surface not only reduces blood cell adhesion but also has the potential to decrease the reliance on anticoagulants, such as heparin, in PDMS-based microfluidic devices. This capability helps maintain high cell viability for target cells. In this study, we used dovetail clips to fix the PDMS and the glass slide together to form the closed microchannels of the PDMS-based microfluidic chips (Figure 5a), reducing anticoagulant use while preventing blood cell adhesion. To simulate a complex physiological environment in vivo, fresh human blood with a low dose of heparin (0.25 U/mL) was introduced into the microfluidic chip to evaluate its anticoagulant performance. The SEM analysis revealed that the liquid-like surface significantly inhibited thrombus formation on its surface. In contrast, the solid surfaces exhibited severe clot formation, including activated platelets, red blood cells, and cross-linked fibrin (Figure 5b). The formation of thrombus may be attributed not only to the low dose of heparin but also to the low flow rate, as the low flow rate generates a small shear force that increases the adhesion of blood cells, thus contributing to thrombus formation [1]. On the liquid-like surface, only a few platelets in a resting, non-activated state were detected (Figure 5c). Thus, the liquid-like interfacial design in a PDMS-based microfluidic device can effectively reduce the need for anticoagulants while preserving antithrombotic properties.

## 4. Discussion

This work proposes a rapid construction method for liquid-like surfaces via single-cycle polymer brush grafting for enhanced antifouling in microfluidic systems. One advantage is that the modification strategy for rapidly constructing liquid-like surfaces is universal. The grafting process involves a ring-opening reaction between succinimide groups and amino groups on the glass surface. For materials, whether organic or inorganic, if there are amino groups on the surface, the liquid-like surface can be successfully modified. Another advantage is that ethanol is used as a green solvent to construct the liquid-like surface by grafting μPDMS. This strategy not only effectively resists cell adhesion but also exhibits superior biocompatibility.

This work not only broadens the versatility of liquid-like surfaces but also lays the foundation for future exploration in a wide range of biomedical applications. For instance, in biosensors, the liquid-like surfaces can improve sensitivity and accuracy by minimizing fouling. Future research could focus on optimizing the grafting process for different materials and applications, exploring the long-term stability and durability of the liquid-like surfaces, and investigating their performance in complex biological environments. This will further expand the applicability of this innovative approach in the biomedical field.

## 5. Conclusions

This study reports a rapid liquid-like surface functionalization strategy by the single-cycle grafting of high-molecular-weight μPDMS on the PDMS surfaces. The incorporation of this liquid-like surface imparts several advantageous properties to the PDMS, including a low sliding angle for blood droplets, excellent antibacterial characteristics, and reduced platelet adhesion. Additionally, the liquid-like surface improves PDMS-based microfluidics by not only diminishing blood cell adhesion and thrombus formation but also minimizing the requirement for heparin. Looking ahead, the liquid-like surfaces, which feature amino groups, offer the potential for further chemical modification or functionalization. For example, it allows for the grafting of specific ligands targeting the epithelial cell adhesion molecule, facilitating the capture of target cells while minimizing non-specific blood cell adhesion. This suggests that the liquid-like surface holds considerable promise for applications in cellular analysis and the assessment of gene or protein expression levels in target cells.

## Data Availability

The original contributions presented in the study are included in the article, further inquiries can be directed to the corresponding authors.
